# Qualitative Descriptive Research Investigating Burn Survivors’ Perspectives on Quality of Care Aspects

**DOI:** 10.3390/ebj5030021

**Published:** 2024-07-01

**Authors:** Raaba S. M. Thambithurai, Lotte van Dammen, Margriet E. van Baar, Hendriët Wanders, Angelique E. A. M. Weel-Koenders, Tsjitske M. Haanstra, Carine M. H. van Schie, Paul P. M. van Zuijlen, Cornelis H. van der Vlies, Eelke Bosma, Corine A. Lansdorp, Inge Spronk, Nancy E. E. Van Loey, on behalf of the National Burn Care, Education & Research Group, The Netherlands

**Affiliations:** 1 Erasmus School of Health Policy and Management, Erasmus University Rotterdam, 3062 PA Rotterdam, The Netherlands; 2 Burn Centre, Maasstad Hospital, 3079 DZ Rotterdam, The Netherlands; 3 Association of Dutch Burn Centres (ADBC), 3079 DZ Rotterdam, The Netherlands; 4 Burn Centre, Martini Hospital, 9728 NT Groningen, The Netherlands; 5 Burn Centre, Red Cross Hospital, 1942 LE Beverwijk, The Netherlands; 6 Department of Public Health, Erasmus MC, University Medical Centre Rotterdam, 3015 GD Rotterdam, The Netherlands; 7 Dutch Association of Burn Survivors, 1941 AJ Beverwijk, The Netherlands; 8 Rheumatology, Maasstad Hospital, 3079 DZ Rotterdam, The Netherlands; 9 Dutch Burns Foundation, 1941 AJ Beverwijk, The Netherlands; 10 Research Group Relational Care, Centre of Expertise Health Innovation, The Hague University of Applied Sciences, 2521 EN The Hague, The Netherlands; 11 Department of Plastic and Reconstructive Surgery, Red Cross Hospital, 1942 LE Beverwijk, The Netherlands; 12 Department of Plastic, Reconstructive and Hand Surgery, Amsterdam UMC, De Boelelaan 1117, 1081 HV Amsterdam, The Netherlands; 13 Pediatric Surgical Centre, Emma Children’s Hospital, Amsterdam UMC, Meibergdreef 9, 1105 AZ Amsterdam, The Netherlands; 14 Amsterdam Movement Sciences (AMS), Tissue Function and Regeneration, 1081 HZ Amsterdam, The Netherlands; 15 Departments of Trauma and Burn Surgery, Maasstad Hospital, 3079 DZ Rotterdam, The Netherlands; 16 Trauma Research Unit, Department of Surgery, Erasmus MC, University Medical Centre Rotterdam, 3015 GD Rotterdam, The Netherlands; 17 Department of Surgery, Martini Hospital, 9728 NT Groningen, The Netherlands; 18 Department of Clinical Psychology, Utrecht University, 3584 CS Utrecht, The Netherlands

**Keywords:** burns, wounds and injuries, quality indicators, health care, patient-centered care, focus group

## Abstract

Burn care quality indicators are used to monitor and improve quality of care and for benchmark purposes. The perspectives of burn survivors, however, are not included in current sets of quality indicators while patient-centred care gains importance. The aim of this study was to explore burn survivors’ perspectives on quality aspects of burn care, which was used to translate their perspectives into patient-centred quality of care indicators. Qualitative descriptive research was conducted in a patient panel group. First, thematic analysis was applied to the focus groups to identify overarching themes. Second, patient-centred quality indicators, informed by burn survivors’ valued aspects of care, were defined. Ten burn survivors with an average age of 54 years (SD = 11; range 38–72 years) and mean TBSA burned of 14% (SD = 11%; range 5–35%) participated in two focus groups. Four overarching themes were identified, pointing to the importance of (1) information tailored to the different phases of recovery, (2) significant others’ wellbeing and involvement, (3) a therapeutic relationship and low-threshold access to healthcare professionals and (4) to participate in decision-making. Eighteen patient-centred process quality of care indicators within nine aspects of care were formulated. The overarching themes are reflected in patient-centred quality indicators, which present a broadened and complementary view of existing clinical quality indicators for burn care. Evaluating these patient-centred quality indicators may increase quality of care and refine patient-centred care.

## 1. Introduction

Severe burn injuries require highly specialised multidisciplinary management in burn centres. Despite increased survival rates, the treatment of severe burns remains challenging from a biopsychosocial perspective to obtain the best results in functioning, scar quality and psychological wellbeing that facilitate social reintegration [1,2,3]. Next to the burn survivor, family members can also be psychologically affected; they are an important source of support necessary to facilitate the recovery process [4,5,6]. All these challenges require timely and adequate management and may differ across the several recovery phases that typically characterise burn care, such as the acute care, sub-acute care and aftercare phases, indicating the dynamics in management from the multidisciplinary team [7]. Optimal treatment of severely burned patients requires the integration of healthcare services and professionals through a multidisciplinary approach. This has led to the development of highly specialised burn centres over the past decades [8]. In the Netherlands, referral criteria to a dedicated burn centre follow EMSB criteria, but less severe burns may also be treated in a burn centre [9]. Patients treated in general hospitals may be referred to a burn care centre at a later stage. Patients with burns in the Netherlands may receive different treatments in various settings and may be faced with different quality of care. 

Measuring and improving quality of care has increasingly gained importance in recent years. Quality indicators are a way to evaluate quality of care and may stimulate quality of care improvements [10,11]. Quality indicators include structure indicators, processes and outcomes of care. Examples of quality indicators are ‘overall length of stay for acute episode of care’ and ‘complications’ [11]. One of the first publications on quality indicators in burn care refers to the Bi-National Burn Registry (Bi-NBR) in Australia and New Zealand [12]. Through the registration of clinical data, quality indicators are included as routine variables. Recently, an update was accomplished, including new quality indicators such as ‘infection control’, ‘pain assessment’ and ‘psychosocial assessment’ [11].

To date, the literature has predominantly focused on quality of care and quality indicators from healthcare professionals’ perspectives [11,12]. Consequently, there is limited evidence on what patients value in their care process, while patients’ perspectives provide unique, complementary and essential information. Quality indicators translated from the perspectives of patients are referred to as ‘patient-centred quality indicators’ [13]. Adding the perspective of burn survivors can contribute to patient-centred care and, in turn, improve recovery [3]. However, translating perspectives of burn survivors into quality indicators is still in its infancy [13]. Aspects of what burn patients value include educational information about treatment and medication, making choices about their treatment, social support of family and friends and peer support during their rehabilitation [14,15].

To the best of our knowledge, there have been no attempts to define patient-centred care needs into quality of care indicators in burn care. Insight into burn survivors’ experiences may help to establish quality indicators reflecting their perspectives. The aim of this study was to explore burn survivors’ perspectives on quality aspects of burn care, which was used to translate their perspectives into patient-centred quality of care indicators.

## 2. Methods

### 2.1. Qualitative Approach

Qualitative descriptive research using thematic analysis was performed to explore how patients perceived the quality of burn care. Qualitative descriptive research can be used when a straightforward description of a phenomenon is desired [16]. This type of research focuses on discovering the nature of specific events without aiming to explain a phenomenon. 

### 2.2. Researchers’ Characteristics

A researcher (NVL, nursing and psychological research background, PhD, female) trained in qualitative research at Utrecht University conducted the focus groups. Two other researchers (LvD, psychological research background, project coordinator research and innovation, PhD, female; CvS, head research and innovation, PhD, female) attended the focus groups to assist with technical issues and to take notes. In the first focus group, a patient experience expert (HW, female) attended the focus group. A PhD student (RT, MSc, researcher economic evaluation), two psychological researchers (LvD and NVL) and an epidemiologist (MvB, PhD, female) were involved in data analysis and discussion of the themes. No relationship was established between the researcher and the participants of the focus groups. Participants only received some background information about the researcher.

### 2.3. Sampling Strategy

In February 2022, 24 burn survivors and parents of children with burns were invited to participate in the focus groups. They are the patient panels from the three Dutch burn centres and can be contacted for questions and meetings that focus on improving burn care. The panels comprise burn survivors varying in burn treatment (i.e., inpatient and outpatient care), time post-burn, burn severity and hospital (i.e., Dutch dedicated burn centres or general hospitals located across the country), but all panel members were treated in a burn centre, be it immediately in the acute phase or at a later phase receiving reconstructive treatments. The patient panel reflects the heterogeneous population of burn patients in the Netherlands. Contact persons of the patient panel first contacted the burn survivors by phone about this research. Hereafter, local researchers sent an email with more information about the purpose of the focus groups to the panel members. They were asked to respond if they were interested in participating. Time constraints were the main reason provided to decline participation. The interested participants were invited to participate in one of two focus groups held on two consecutive days. They could indicate which day they preferred. To avoid travel time, the focus groups were conducted online.

### 2.4. Data Collection

The lived experiences regarding which aspects of care were valued were explored in a convenience sample [17]. The focus groups were semi-structured by an unpiloted interview guide and explored three phases: (1) admission to the hospital and, for some, subsequent admission to the burn centre; (2) the period of hospitalisation and (3) the aftercare period. For every phase, the questions were open and broad and explored what was important for the patients, such as ‘What was important to you when you were admitted to the burn centre?’ (Appendix A). More detailed information was obtained by using probe questions. Quality indicators described by Gong et al. were questioned if indicated [11]. The focus groups were held via Zoom in March 2022. These Zoom sessions were audio recorded and transcribed verbatim.

### 2.5. Data Analysis

The transcribed data were imported into MAXQDA 2020 software and analysed inductively using thematic analysis [18]. Three researchers (RT, LvD and NVL) independently analysed and coded the data line-by-line. Two researchers (RT and LvD) discussed similarities and differences and reached a consensus on the codes, which were merged into a code tree. Together with the third researcher (NVL), triangulation of data was achieved, which reduced the potential for researcher bias. Final codes were merged into a final code tree after several discussions with four researchers (RT, LvD, NVL and MvB). During these discussions, themes about what was important for the participants during their care process were established. In a second analysis, the transcripts were re-read and re-analysed deductively to define patient-centred quality of care indicators, mainly focussed on processes. To define a patient-centred quality indicator, valued aspects of care were identified and included as a quality indicator. In the next step, the patient-centred quality indicators were compared to clinical quality indicators described by Gong et al. [11].

### 2.6. Ethical Aspects

This study was conducted in accordance with the Declaration of Helsinki [16]. The Medical Research Ethics Committees United (MEC-U) judged that this study (number W21.305) did not fall under the scope of the Dutch Medical Research Involving Human Subjects Act (WMO). Informed consent was obtained from all study participants.

## 3. Results

### 3.1. Participant Characteristics

Ten of the twenty-four invited burn survivors and parents of children with burns participated in the focus groups, including nine burn survivors and one mother of a paediatric patient. The focus groups included seven (transcript 1) and three (transcript 2) participants, respectively. The focus groups lasted 120 and 100 min, respectively. Demographic and injury characteristics of participating patients are presented in Table 1. Most of the participants had a length of stay of less than one month, with a range of 3–87 days. One participant had a length of stay of 87 days due to major burns. The TBSA burned was between 5% and 35%. Only two participants had a TBSA burned >10%. The age range was between 38 and 72 years. Given the variation in burn severity, not all participants underwent surgery. 

### 3.2. Burn Survivors’ Perspectives on Important Aspects of Care

Thematic analysis resulted in four overarching themes describing burn survivors’ perspectives about what they consider important during the various recovery phases: (1) the importance of information tailored to the different phases of recovery, (2) the importance of significant others’ wellbeing and involvement during the recovery, (3) the importance of a therapeutic relationship and low-threshold access to healthcare professionals to ensure care continuity and (4) the importance of participation in decision-making.

#### 3.2.1. The Importance of Information Tailored to the Different Phases of Recovery

During the different phases of recovery, participants had a strong desire for information, which was described as ‘information hunger’. In all phases, they were eager to know why procedures were carried out and expressed the desire to be informed about what to expect in terms of outcomes. They recommend tailoring information to the different phases and dose information to keep up their spirits.

*‘I was very hungry for information, and really wanted to know what was going to happen to me and when.’ (Transcript 1, outpatient)*

*‘On the one hand you want information, but also it was better not to know everything. That makes it possible to stay positive. So, I think it might better not to know everything at once.’ (Transcript 2)*

Participants who were sent home after they received first aid and had an outpatient trajectory reported that they liked to be informed about possible complications, such as fever, and issues like pain medication, prognosis and timing of the surgery.

*‘I would have preferred to know what was happening, what the plan was and what was going to happen in the next week or two.’ (Transcript 1, outpatient)*

The outpatients searched the internet for information in the acute phase, while inpatients often did this after discharge. However, participants reported it was still difficult to find reliable information or to find all the information they were specifically looking for. Although they understood that not all the answers were immediately available, these uncertainties could provoke anxiety and distress. The Dutch aftercare site, promoted after discharge by burn care staff, was considered a useful tool for finding easily accessible information.

*‘For example, I could find information about grafts, but what would the scar look like and what sort of time period were we talking about?’ (Transcript 1, outpatient)*

*‘So this aftercare site is also easily accessible for everyone when it best suits them. I think it would be very useful to improve this.’ (Transcript 2)*

Regarding inpatients, participants felt relieved when they were admitted to a burn centre because they felt they were in good hands. In the early admission phase, they wanted to understand the severity of their burns, whereas, during their stay at the burn centre, the need for information shifted towards information on burn wound depth and its immediate consequence (i.e., whether a surgical intervention would be needed). When surgery was indicated, participants recommended being informed about aspects of surgery that would cause discomfort, like staples.

*‘In the beginning, I think I needed more insight into what was happening. When is it decided if someone needs surgery or not.’ (Transcript 1)*

*‘What could I roughly expect? For example, that first of all the wound would be left to settle down, I think I needed that sort of information, what it would be like. When would they decide to operate or not?’ (Transcript 1)*

Relating to the use of skin substitutes, participants expressed the importance of being informed about safety issues, the healing process and long-term scar results.

*‘I think if you get a skin substitute, you want to know what the pros and cons are. What makes a skin substitute better than your own skin?’ (Transcript 2)*

*‘What is most important, is the healing process and how it [the scar] is going to look like.’ (Transcript 1)*

In the aftercare phase, participants like to be informed about how scars evolve and when aesthetic issues, such as redness and discomfort, and functioning, such as stiffness, improve.

*‘My only complaint, what I kept trying to find out, is how long will my hands stay red? How long will this stiffness last? Because I keep trying to move my fingers a bit more so that they move normally.’ (Transcript 2)*

In the aftercare phase, participants reported that it was difficult to anticipate their care needs after discharge and recommended getting tips and tricks to deal with discomfort.

*‘In hospital they knew how to help, for example on how to get comfortable when lying down. It would have been useful to be better prepared for the situation at home, with tips and tricks, you know.’ (Transcript 1)*

Participants indicated that peer support could be helpful in coping with the consequences of burns. Receiving information from people with similar experiences was perceived as helpful in understanding the recovery process.

*‘I think it helps to speak with someone who has also experienced this. It is easier to listen to them than to people who have not actually experienced it themselves.’ (Transcript 2)*

*‘The experiences of others; what will it look like in a couple of months, what should you watch out for, what are the pitfalls. Those are the sorts of things it is important to know.’ (Transcript 2)*

#### 3.2.2. The Importance of Significant Others’ Wellbeing and Involvement during the Recovery

Participants expressed concerns about the wellbeing of their loved ones (i.e., significant others). During their admission, there was a strong desire to contact direct family members to assure them they were in good hands. Participants appreciated that significant others were cared for, for example, the availability of psychological care and tailored visiting hours and arrangements were highly appreciated.

*‘I didn’t have difficulties with emotional problems and still don’t. But my wife had. She saw it [the burn event] all happening and saw how miserable I was. And she was given excellent support [by the burn care staff].’ (Transcript 2)*

*‘What I heard later is that my dearest friends and family were really well looked after. Time was taken to explain things to them, also by a psychologist. I am very grateful that they were so well looked after.’ (Transcript 2)*

*‘It happened during COVID-19 time. Only one visitor per day was allowed. When I had the accident I ran around like a mad thing. My young children saw me in the shower, with skin hanging off me. I said [to the burn care staff] how much it meant for them [my children] to be able to visit me. […] So it was arranged that they visited one at a time. I was very grateful that this was possible, even though it meant not strictly following the rules.’ (Transcript 2)*

During their stay at the burn centre, participants mentioned the importance of the involvement of the family during the recovery. The partner and parents can play a part in performing tasks that are in the patient’s interest. For example, the family of a ventilated patient was advised to keep a diary, which helped the participant in a later phase of recovery to understand what happened during the admission. A parent expressed the need to stay in the burn centre with their child, including during care procedures, in order to take care of and comfort the child. Participants also valued the presence of their partner when they were informed about treatment issues.

*‘But if you are going to get surgery with donor skin or a skin graft, […] or whatever, it is very important that your partner is present [when information is provided].’ (Transcript 1)*

After discharge, some participants expressed that their partner was involved in care procedures. It was found helpful that patients and partners were prepared in the burn centre to perform wound care at home.

*‘My wife was present during the last wound care treatment in the hospital and everything was explained to her so that she knew what to do when we were at home.’ (Transcript 1)*

#### 3.2.3. The Importance of a Therapeutic Relationship and Low-Threshold Access to Healthcare Professionals to Ensure Care Continuity

Participants valued therapeutic relationships (e.g., active listening, empathy and meeting the needs of the patient) with healthcare professionals during admission. During their stay in the burn centre, they appreciated small talk with healthcare professionals. It helped them to build a relationship with the staff and made it easier to share their concerns, for example, the need for more psychological care.

*‘The doctor came in the morning, […]. What I also really appreciated was when one of the junior doctors spend a bit more time with me. When I said, for example, that I was concerned about my second child, they said that a social worker could be arranged to speak with her next time she [child] visited. […] Also, I had easy access to psychological support and that was very helpful.’ (Transcript 2)*

After discharge, low-threshold access to healthcare professionals (e.g., the aftercare nurse) was important for solving problems they encountered. Participants valued that nurses were direct and easy to reach and took the time to talk to patients by telephone or digitally to solve problems they were faced with at home. They advocated that the use of digital support could even be intensified because it allowed real-time showing of the problem.

*‘Convalescence begins the moment you leave the hospital. In hospital everything is available and organised for you. At home it is quite different. Then it is very comforting to know there is a backup.’ (Transcript 1)*

*‘I think that the digital aspect could be improved so that it would be possible to make a better digital assessment. I would recommend digital video contact so that the nurse can see it [wounds].’ (Transcript 1)*

#### 3.2.4. The Importance of Participation in Decision-Making

Participants valued being involved in decision-making, for example, regarding pain medication. Another participant expressed the desire for collaborative discharge planning (e.g., related to phasing out pain medication). Factors that influenced the desire to be involved in decisions differed across participants but were largely related to long-term outcomes and side effects.

*‘Actually, I expected the nurses to suggest that, as I would be going home soon, it would be better to see how I could manage with less painkillers.’ (Transcript 1)*

### 3.3. Patient-Centred Quality of Care Indicators

Participants’ opinions about aspects of care were summarised and compared to internationally defined clinical quality indicators [11]. Gong et al. included fourteen process quality indicators within ten aspects of care. Our summary resulted in eighteen patient-centred quality of care indicators within nine aspects of care. A comparison of our study with Gong et al. resulted in five overlapping aspects of care. Ten patient-centred process quality indicators were identified within these five overlapping aspects of care. Patient-centred indicators not comprised in the existing clinical process quality indicators from Gong et al. were identified when participants had indicated this as valuable aspects of care. This resulted in eight patient-centred process indicators within four new valuable aspects of care, presented in Table 2.

## 4. Discussion

This study explored burn survivors’ perspectives about what they valued during their care process in a burn centre and after discharge. It revealed four overarching themes that are reflected in the patient-centred process quality indicators. When comparing these patient-centred quality indicators with existing clinical quality indicators, there is considerable overlap, and they present an additional and complementary view of quality of care. Moreover, beyond the existing quality indicators, this study identified new quality indicators that may broaden the view on quality of care from the patient’s perspective and thereby improve quality of patient-centred care.

Participants in this study expressed the importance of information, which was crucial for the understanding of the care process and long-term expectations and to regulate negative emotions, such as anxiety and distress. These findings corroborate existing studies that identified the importance of clear health information, such as educational and preparatory information during the acute phase, and more actionable and user-friendly information in the longer term [15,19]. In line with other studies, burn survivors want to receive information about, for example, sleep and pain medications, risks of side effects, alternative treatments, recovery expectations, scar management, available mental health support and future treatment plans [2,20]. Overall, information about different aspects of care is of importance to burn survivors in all phases of their recovery and, therefore, can be found in several patient-centred quality indicators.

Furthermore, this research showed that healthcare providers, the internet and other burn survivors act as a source of information. As earlier reported, healthcare professionals were considered a source of reliable and personalised information about treatment options and the progression of recovery and to validate the information gained from other sources [19]. In our study, outpatients searched the internet for information about what to expect in the acute phase, while others used it after discharge to learn more about scar maturation but found it difficult to find information applicable to their situation. This is in line with a study reporting that the internet was used to find general information about burns and treatment options but often insufficiently addresses long-term effects, peer experiences and information about discharge and rehabilitation phase [19]. This encourages the further development of patient-centred public web pages in which personalised information is pursued. In line with our study, peer support was a source of empowerment as it gives hope and confidence to burn survivors and provides additional information [19,21,22]. This subscribes to the relevance of peer support activities already available all over the world.

Our study suggests that the role of significant others was important in the recovery process, and burn survivors were concerned about their wellbeing. Burn survivors expressed the desire to involve significant others in decision-making, for example, related to surgical intervention and their involvement in care procedures during the acute phase and thereafter when, for example, wound care procedures are still required. The importance of significant others in the recovery process is well-established [23]. Several studies also point to the importance of the involvement of significant others when receiving information or during care procedures [24,25]. Significant others, however, may be psychologically affected by the burn event themselves while being an important source of support at the same time [26]. Burn survivors appreciated that their significant others received psychological support, indicating their concern for their significant others’ wellbeing. This was evident immediately after the burn event and continued throughout admission. Given the central role of significant others in supporting the burn survivor throughout the recovery process, psychological support for significant others was incorporated as a patient-centred quality indicator.

The therapeutic relationship and low-threshold access to healthcare professionals were found important during admission and after discharge. Building a therapeutic relationship involves active listening, empathy, creating a safe environment and meeting the needs of the patient [27]. Another study reported that burn survivors cherish having healthcare professionals who listen, understand and care about them and have small talk with them [15]. In our findings, therapeutic relationships helped burn survivors share their concerns and resolve problems and their needs, which was also described in a literature review [28]. Furthermore, burn survivors advocate smooth care transitions and low-threshold contact. Patients want clear information about who is responsible for overseeing their care and care continuity and clarity about who the first point of contact is to call for advice [29]. In general, patients valued follow-up calls and a contact person to call after discharge to check in with them [30]. The use of telehealth services in burn care provides a possibility for video consultancy for burn survivors during the recovery phases [31,32]. Telehealth is a way to continue follow-up care for burn survivors and to provide low-threshold access to multiple healthcare professionals in the burn centre [32].

Being involved in decision-making was found important, for example, regarding pain interventions. This supports other studies on burn survivors [15,33], which is considered a sign of respecting patients’ autonomy. Offering choices entails providing adequate information [33], which is a prerequisite for shared decision-making (SDM) and facilitates patients to explore preferences based on their own values and beliefs [34]. The involvement of patients in healthcare decision-making has been shown to improve health services and outcomes [35]. Studies showed that patient-centred care, in general, leads to higher quality of care and greater quality of life [36].

The information derived from this research about the patient’s perspective on valued care was translated to quality of care indicators, mainly process indicators, and revealed a broadened and complementary view of clinical quality indicators. Additional aspects of care valued by patients were also translated into patient-centred quality of care indicators. More particularly, tailored communication and the involvement of significant others in care decision-making can optimise quality of care. Another study showed that, in general, good communication with healthcare professionals was an indicator of quality of care [37], supporting our findings. Interestingly, a large range of established clinical indicators was mentioned by burn survivors, validating the professional’s view of quality of care aspects. By adding the patient’s perspective, a more comprehensive view on quality of care can be achieved. However, further elaboration of patients’ perspectives on indicators of quality of care in burn care is still needed as this study was the first exploration in this underdeveloped area. The perspective of burn survivors and the clinical perspective on indicators of quality of care are complementary and mutually reinforcing, providing a new perspective on clinical quality indicators.

A strength of this study is that it is the first to explore patient-centred quality of care indicators. This suggests that professional clinical quality indicators are also of importance to burn survivors and provide a complementary view of what healthcare professionals have already identified. It aligns with standardised patient-centred outcome measures that are increasingly used to measure care outcomes and move beyond this practice by formulating patient-centred quality indicators. This study has several limitations that should be noted. First, this study used convenience sampling useful for pilot testing but limits the ability to generalise. The cohort consists of participants who had a relatively high age, most had a TBSA burned <10%, and not all participants underwent surgical procedures. Moreover, paediatric and young adults were not represented, and only one parent was included. Additionally, the panel did not comprise patients from minority groups who may value other aspects of care (e.g., relating to communication) [38]. Consequently, their perspectives are not represented. All this may impact the generalisability of this study. However, the sample showed variation in injury-related characteristics and provided in-depth information about a range of aspects that corroborate themes reported in earlier studies, as discussed above. Second, the size of one of the focus groups was large (n = 7), given the online conduction. Consequently, not all participants may have had enough time to report their experiences on all the topics, which may have influenced the data collection. Moreover, the transcripts were not returned to participants, and they did not provide feedback on the findings. Third, the number of focus groups was probably insufficient to reach data saturation. It has been established that two to three focus groups are needed to identify 80% of the themes [39]. Possibly related to this issue, not all clinical quality indicators reported by Gong et al. were considered and discussed by participants. However, four themes that align with the literature were clearly identified. Methods to promote credibility such as member checking and coder triangulation were performed. The study question is broad and further granular themes might come from additional focus groups. Future studies may explore valued care in larger samples, including patients with different backgrounds, and focus on outcome indicators and process indicators in order to broaden the scope of patient-centred quality indicators.

In conclusion, this study was the first explorative attempt to investigate patients’ perspectives on valuable care. The need for information tailored to the different phases of recovery, involvement of significant others during the recovery, therapeutic relationship and low-threshold access to healthcare professionals, and the desire to participate in decision-making were found important. These themes were reflected in the patient-centred process quality of care indicators that complement existing clinical quality indicators. This provides a promising avenue for the further development of indicators. Further research on perceived valuable care of burn survivors and how to integrate these values into patient-centred quality indicators is needed. Moreover, more research is needed into the use of patient-centred and clinical quality indicators in practice. This may inform, be used to evaluate and improve quality of burn care and, ultimately, may refine patient-centred care.

## Figures and Tables

**Table 1:** Demographics of participants and injury characteristics.

Characteristics	Total Sample (*n* = 7) ^1^
Males, *n* (%)	5 (48%)
Current age ^2^ (years), mean (SD)	54 (11)
Length of hospital stay (days), mean (SD)	29 (28)
%TBSA burned ^3^, mean (SD)	14 (11)
Surgery (yes), *n* (%)	5 (71%)

^1^ Characteristics of three patients are missing since sharing information was voluntary. ^2^ Age of one participant is missing. ^3^ TBSA data of one participant are missing.

**Table 2:** Clinical process quality indicators compared to patient-centred process quality indications.

Aspects of Care	Clinical Quality Indicators According to Gong et al. [11] ^1^	Patient-Centred Quality of Care Indicators According to This Study
First aid	Was any first aid applied? If yes: Was the first aid applied 20 min of cool running water within three hours of injury?	Were significant others informed? Were procedures and severity of injury explained?
Pain assessment	Did the patient have a pain assessment completed (using a validated pain scale) within 24 h of admission?	Was pain medication evaluated?
Burn wound assessment	Was the burn size documented? Assessment date/time Who completed the assessment?	Was burn severity and expected treatment clearly and timely explained to the patient?
Psychosocial assessment	For patients with an LOS exceeding 48 h, did they have their psychosocial needs screened during their admission? For patients who tested positive in their psychosocial screen, were they referred to psychosocial services within 24 h of the positive screen? When did psychosocial assessment occur?	Were psychosocial services available to patients during admission? Were psychosocial services available to family during admission? Were psychosocial services available to patients after hospital discharge? Were psychosocial services available to family after hospital discharge?
Excision of deep burns	What date was the deep burn excision completed?	Was the timing of surgery communicated? Was the procedure of surgery explained (e.g., use of staples)?
Use of skin substitutes	Not applicable ^2^	Were safety issues of skin substitutes explained to the patient? Was the healing process and long-term scar results of skin substitutes explained to the patient?
Partner and/or family	Not applicable ^2^	Were the patient and significant others offered the possibility to be informed and involved in decision-making? Were significant others prepared and given instructions for care at home?
Aftercare	Not applicable ^2^	Was the patient informed about the availability of an aftercare nurse for questions after hospital discharge? Was the patient informed about where to find digital information?
Contact between patients/burn survivors	Not applicable ^2^	Was the patient informed about contacting other burn survivors during the admission period? Was the patient informed about contacting other burn survivors after hospital discharge?

^1^ The following clinical process quality indicators of Gong et al. were not a topic of discussion in our study: length of stay, physical functioning assessment, enteral/parenteral feeding, malnutrition risk screening and assessment, fluid resuscitation. ^2^ In Gong et al., this topic was not included as a clinical process quality indicator.

## Data Availability

The original contributions presented in the study are included in the article, further inquiries can be directed to M.E. van Baar (data@burns.nl).

## Appendix A. Topic List ‘Focus Group Quality Indicators’

General questions were repeated for the acute phase (pre-hospitalisation and hospitalisation) and sub-acute phase

- What were your experiences, and what was important?
- What did you need at that specific phase?
- What went well and what went not so well?

Specific topics of interest

- Surgery: Were you informed about surgery? How did that go, and what did you need? When?
- Was there consultation about the operation itself or whether you were eligible for an operation with skin substitutes?
- Skin substitutes: What would be important to agree with the application of skin substitutes? (you can think of better scar quality, but two operations are required).
- Wound care: What was important related to wound care?
- Pain: What was important related to pain measurement and pain treatment?
- Psychosocial care: Was psychosocial care provided, and what was important?

Specific topics of interest related to discharge and aftercare

- What were your experiences related to discharge (preparation)? What was important?
- What were your experiences related to aftercare? What was important?
- What were your experiences with outpatient aftercare?
- Contact with healthcare workers
